# *PiggyBac* Transposon-Mediated CD19 Chimeric Antigen Receptor-T Cells Derived From CD45RA-Positive Peripheral Blood Mononuclear Cells Possess Potent and Sustained Antileukemic Function

**DOI:** 10.3389/fimmu.2022.770132

**Published:** 2022-01-27

**Authors:** Masaya Suematsu, Shigeki Yagyu, Nobuyoshi Nagao, Susumu Kubota, Yuto Shimizu, Miyuki Tanaka, Yozo Nakazawa, Toshihiko Imamura

**Affiliations:** ^1^Department of Pediatrics, Kyoto Prefectural University of Medicine, Graduate School of Medical Science, Kyoto, Japan; ^2^AGC Inc. Innovative Technology Laboratories, Yokohama, Japan; ^3^AGC Inc. Materials Integration Laboratories, Yokohama, Japan; ^4^Department of Pediatrics, Shinshu University School of Medicine, Nagano, Japan

**Keywords:** CAR-T cells, CD45RA, CAR-T cell therapy, *piggyBac* transposon, naïve/stem cell memory-like T cells

## Abstract

The quality of chimeric antigen receptor (CAR)-T cell products, namely, memory and exhaustion markers, affects the long-term functionality of CAR-T cells. We previously reported that *piggyBac* (PB) transposon-mediated CD19 CAR-T cells exhibit a memory-rich phenotype that is characterized by the high proportion of CD45RA^+^/C-C chemokine receptor type 7 (CCR7)^+^ T-cell fraction. To further investigate the favorable phenotype of PB-CD19 CAR-T cells, we generated PB-CD19 CAR-T cells from CD45RA^+^ and CD45RA^−^ peripheral blood mononuclear cells (PBMCs) (RA^+^ CAR and RA^−^ CAR, respectively), and compared their phenotypes and antitumor activity. RA^+^ CAR-T cells showed better transient gene transfer efficiency 24 h after transduction and superior expansion capacity after 14 days of culture than those shown by RA^−^ CAR-T cells. RA^+^ CAR-T cells exhibited dominant CD8 expression, decreased expression of the exhaustion marker programmed cell death protein-1 (PD-1) and T-cell senescence marker CD57, and enriched naïve/stem cell memory fraction, which are associated with the longevity of CAR-T cells. Transcriptome analysis showed that canonical exhaustion markers were downregulated in RA^+^ CAR-T, even after antigen stimulation. Although antigen stimulation could increase CAR expression, leading to tonic CAR signaling and exhaustion, the expression of CAR molecules on cell surface after antigen stimulation in RA^+^ CAR-T cells was controlled at a relatively lower level than that in RA^−^ CAR-T cells. In the *in vivo* stress test, RA^+^ CAR-T cells achieved prolonged tumor control with expansion of CAR-T cells compared with RA^−^ CAR-T cells. CAR-T cells were not detected in the control or RA^−^ CAR-T cells but RA^+^ CAR-T cells were expanded even after 50 days of treatment, as confirmed by sequential bone marrow aspiration. Our results suggest that PB-mediated RA^+^ CAR-T cells exhibit a memory-rich phenotype and superior antitumor function, thus CD45RA^+^ PBMCs might be considered an efficient starting material for PB-CAR-T cell manufacturing. This novel approach will be beneficial for effective treatment of B cell malignancies.

## Introduction

Although chimeric antigen receptor (CAR)-T cell therapies targeting CD19 have achieved spectacular success for B-cell malignancies, long-term remission occurs only in half of the patients with B-cell malignancies (1–6). Therefore, enhancing the long-term functionality of CAR-T cells without affecting their anti-tumor potency is important. Although the antitumor efficacy of CAR-T cells depends on various host factors, namely, the disease status or actively hostile tumor microenvironment, the recent clinical studies on CAR-T cell therapies have reported that the quality of CAR-T cell products, namely, T-cell memory signatures and exhaustion-related markers, is critical for the function and antitumor efficacy of CAR-T cell therapies (7–9). The presence of naïve and stem cell memory-like CAR-T cells in the final product is correlated with the response of CAR-T cell therapies in B-cell malignancies (7). Therefore, the manufacturing process of CAR-T cells should be optimized to prevent early T-cell exhaustion and to maintain the memory phenotype during the expansion step. Pre-activation of T cells by anti-CD3 and CD28 antibodies—an indispensable step for retroviral or lentiviral gene transfer into T cells—strongly induces T-cell differentiation and exhaustion; therefore, various efforts to obtain memory-rich CAR-T cells have been attempted, namely, the use of interleukin (IL)-7 and IL-15 cocktails instead of IL-2 or transient stimulation with anti-CD3 and CD28 antibodies (7, 10).

Non-viral gene transfer using *piggyBac* (PB) transposon-based genetic modifications is a potentially effective strategy for CAR-T cell manufacturing (11–16). Our previous study and also other studies have reported that PB-CAR-T cells exhibit an enriched memory fraction and less exhaustion-related markers, regardless of the type of CAR constructs, electroporation conditions, or expansion protocols (17, 18). PB-CAR-T cells that redirected CD19, HER2, or ephrin type-B receptor 4 precursor (EPHB4) molecules were dominant in the naïve/stem cell memory-like T-cell fraction, which was characterized as CD45RA/C-C chemokine receptor type 7 (CCR7) double positive and is related to long-term functionality.

As the nature of T cells in the starting peripheral blood mononuclear cell (PBMC) materials affects the phenotype and function of CAR-T cells in the final product (19), the composition of the starting PBMC materials would contribute to the maintenance of the memory phenotype of PB-CAR-T cells in the final product. The phenotype of patient PBMCs might be greatly affected by other diseases or prior chemotherapeutic agents (19, 20), and is associated with the manufacturing success rate, phenotype, and functionality of autologous, patient-derived CAR-T cell products. Therefore, the optimization of the composition of starting PBMC materials would be important for the stable manufacturing of memory-rich PB-CAR-T cells. To improve the manufacturing success rate and the functions of CAR-T cells, previous studies have reported that the enrichment of whole T cells by elimination of monocytes and granulocytes from starting materials would improve T-cell activation and transduction efficiency during virally-engineered CAR-T cell processing (21–23); however, optimal PBMC subpopulations for PB-CAR-T cell processing are unknown. In this study, we aimed to generate PB-CD19 CAR-T cells using the subpopulations of PBMCs based on CD45RA expression and to investigate the usefulness of CD45RA positive PBMC subpopulation as the starting material for PB-CD19 CAR-T cell manufacturing.

## Materials and Methods

### Ethics Approval and Consent to Participate

This study was approved by the Institutional Review Board of Kyoto Prefectural University of Medicine (Approval Numbers: ERB-C-669 and ERB-C-1406) and the recombinant DNA experiments were approved by the safety committee of the recombinant DNA experiment of Kyoto Prefectural University of Medicine (Approval Numbers 2019-111 and 2019-112). All experiments involving human participants were performed in accordance with the Declaration of Helsinki guidelines. All animal experiments and procedures were approved by the Kyoto Prefectural University of Medicine Institutional Review Board (Permit No.: M2020-13).

### Blood Donors and Cell Lines

Blood samples from healthy donors were obtained with a written informed consent, and PBMCs were isolated from the whole blood samples by density gradient centrifugation using Lymphocyte Separation Medium 1077 (FUJIFILM Wako Pure Chemical Corporation, Osaka, Japan), followed by multiple washes with Dulbecco’s phosphate-buffered saline (D-PBS; Nakarai Tesque, Kyoto, Japan). The number of live cells was determined by standard trypan blue staining and using an automated cell counter model R1 (Olympus, Tokyo, Japan). The human lymphoblastic leukemia cell line (REH) was purchased from the American Type Culture Collection (Manassas, VA). REH-expressing firefly luciferase (FFLuc) and green fluorescent protein (GFP) (REH-FFLuc-GFP) were obtained by introducing PB-based pIRII-FFLuc-puroR-GFP (18) in REH cells and subsequent fluorescent-activated cell sorting. REH and REH-FFLuc-GFP cells were cultured in Roswell Park Memorial Institute-1640 medium (Nacalai Tesque) supplemented with 10% fetal bovine serum (Thermo Fisher Scientific, Inc. Waltham, MA) and maintained in a humidified incubator at 37°C in a 5% CO_2_ atmosphere.

### Plasmid Construction

The PB transposase plasmid, pCMV-piggyBac (24), which contained ~2.4 kb of transposase elements with identical 13 base pair (bp) terminal inverted repeats and additional asymmetric 19 bp internal repeats (25, 26), was artificially synthesized (Mediridge Co., Ltd, Tokyo, Japan). CAR construct for CD19− CAR-T cells which encodes the CD19 specific scFv, followed by a short hinge, the transmembrane and signaling domain of the costimulatory molecule CD28, and the ζ signaling domain of the TCR complex, was kindly provided from Dr. Cliona M. Rooney (Baylor College of Medicine) and was subcloned into pIRII transposon vector backbone (11) (pIRII-CD19-28z) as described previously (27). For the generation of antigen-presenting feeder cells for the stimulation of CD19-CAR-transduced T cells, we used a plasmid containing sequences encoding the extracellular, transmembrane, and 20 amino-acid-long intracellular portion of CD19 protein (tCD19) driven by CMV promoter, followed by CD80 and 4-1BBL (CD137L) with TA2 and P2A self-cleaving sites that enabled independent gene expression. The tCD19-CD80-4-1BBL sequence was artificially synthesized (Fasmac Inc., Kanagawa, Japan) and cloned into a pIRII PB transposon vector (pIRII-tCD19-CD80-4-1BBL) (**Supplementary Figure S1**) (17, 18).

### Manufacturing of PB-Mediated CAR-T Cells

CD45RA^+^ and CD45RA^−^ PBMCs were isolated by magnetic selection from the whole PBMCs using CD45RA MicroBeads, human (Miltenyi Biotec, Bergisch Gladbach, Germany) (**Supplementary Figure S2A**). The CD19-CAR transgene was then transduced into these cells using the PB transposon system, as described previously (17, 18). Briefly, pCMV-piggyBac (7.5 μg per 100 µl of electroporation buffer) and pIRII-CD19-28z (7.5 μg per 100 µl) (**Supplementary Figure S1**) were introduced into about 4 × 10^6^ CD45RA^+^ or CD45RA^−^ PBMCs, respectively, using the P3 Primary Cell 4D-Nucleofector™ X kit (Lonza, Program; FI-115) or MaxCyte ATX^®^ (MaxCyte Inc) with the optimized protocol for introduction of DNA plasmid into resting T cells (Protocol; RTC 14-3). Concurrently, an antigen-presenting feeder plasmid (pIRII-tCD19-CD80-41BBL; 15 μg per 100 µl) (**Supplementary Figure S1**) was introduced into approximately 1 × 10^6^ whole PBMCs by electroporation. After electroporation, the CAR-T cells and feeder cells were cultured in complete culture medium consisting of ALyS™705 Medium (Cell Science & Technology Institute) supplemented with 5% artificial serum (Animal-free; Cell Science & Technology Institute), IL-7 (10 ng/ml; Miltenyi Biotec), and IL-15 (5 ng/ml; Miltenyi Biotec). The feeder cells were irradiated with ultraviolet light for inactivation 24 h after electroporation and co-cultured with CAR-T cells for 14 days, as described previously (17, 18). CAR-T cells redirected to the EPHB4 receptor were manufactured using the PB transposon system as described previously (17) for its use as the control CAR-T cells in the *in vivo* stress test (**Supplementary Figure S1**).

### Flow Cytometry

Expression of CD19-CAR molecules on T-cell surface was measured by flow cytometry using the recombinant human CD19 Fc chimera protein (R&D Systems, Minneapolis, MN, USA) and goat anti-human immunoglobulin (Ig)-G Fc fragment specific antibody conjugated to fluorescein isothiocyanate (FITC) (Merck Millipore, Burlington, MA). Allophycocyanin (APC) or phycoerythrin (PE)-conjugated anti-CD3 antibody, FITC-conjugated anti-CD19 antibody, PE-conjugated anti-CD56 antibody, FITC-conjugated anti-CD15 antibody, APC-conjugated anti-CD14 antibody, APC-conjugated anti-CD8 antibody, PE-conjugated CD4 antibody, PE-conjugated anti-CD45RA antibody, and APC-conjugated anti-CCR7 antibody (all from BioLegend, San Diego, CA, USA) were used to characterize the phenotypes of CAR-T cells. APC-conjugated anti-programmed cell death protein-1 (PD-1) antibody, APC-conjugated anti-T cell immunoglobulin mucin-3 (TIM-3) antibody, Alexa Fluor 647-conjugated anti-CD223 (LAG-3) antibody, and Peridinin-Chlorophyll-Protein (PerCP)/Cyanine5.5-conjugated anti-CD57 antibody were used as the exhaustion and senescence markers of CAR-T cells (all from BioLegend). FITC-conjugated anti-CD19 antibody was also used to determine the phenotype of REH cells. All flow cytometry data were acquired using BD Accuri™ C6 Plus or BD FACSCalibur™ (BD Biosciences, Franklin Lakes, NJ) and analyzed using the FlowJo™ software (BD Biosciences).

### Transgene Copy Number Analysis

After 14 days of culture, 1 × 10^5^ CAR-positive T cells were isolated using a Cell Sorter SH800 (SONY, Tokyo, Japan), and total DNA was then extracted using a QIAamp DNA Mini Kit (QIAGEN, Hilden, Germany). Quantitative PCR was carried out using the total DNA from 1 × 10^3^ CAR-positive T cells (equivalent to 1 µl of DNA extract) and custom primer/Taqman probe set specific for the CD19-CAR transgene at the junction of CD28 cytoplasmic domain and CD3ζ by a 7500 Real-Time PCR System (Applied Biosystems, Foster City, CA, USA). To measure DNA copy number for absolute quantification, pIRII-CD19-CAR plasmid was used (**Supplementary Figure S3A**). The relevant primers and Taqman probe are shown (**Supplementary Table S1**).

### Analysis of Exhaustion-Related Markers Expressed on T Cell Surface After Electroporation

The GFP plasmid was introduced into the whole PBMCs by electroporation using the same protocol as that used for CAR-T manufacturing. After electroporation, the GFP-introduced PBMCs were cultured in complete culture medium consisting of the same components used for CAR-T manufacturing. PBMCs without electroporation were cultured in the same medium and was used as the control. After 48 h of incubation, the expression of PD-1, TIM-3, and LAG-3 on T cell surface was analyzed by flow cytometry by gating GFP-positive and CD3-positive T cells.

### Sequential Killing Assay

We co-cultured 1 × 10^5^ REH cells and 1 × 10^5^ CD19 CAR-T cells derived from CD45RA^+^ or CD45RA^−^ PBMC (RA^+^ CAR or RA^−^ CAR, respectively) in 24-well cell culture plates. Three days later, the CD19 CAR-T cells were collected, counted, and treated and reconstituted with fresh REH cells at a ratio of 1:1. Cell counting and treatment with fresh REH cells were repeated every three days for a total of three iterations. The killing effect of these CD19 CAR-T cells was evaluated by counting the number of residual REH cells by flow cytometry. The mean fluorescence intensity (MFI), exhaustion-related markers, cytokine production, and proliferation of the CAR-T cells were analyzed by flow cytometry.

### Cytokine Production Assay

The levels of interferon (IFN)-γ, tumor necrosis factor (TNF), and IL-6 were measured using a Cytometric Bead Array (CBA) Kit (BD Biosciences). Briefly, CAR-T cells were co-cultured with tumor cells at a ratio of 1:1. After 3 days of co-culture, the cell culture supernatant was collected and cytokine levels were determined and analyzed. Data were acquired with a BD Accuri C6 Plus (BD Biosciences) and analyzed with FCAP Array v.3.0 (BD Biosciences).

### CytoTell™ Dilution Assay

We examined the proliferation of CAR-T-cells using CytoTell™ Red 650 (AAT Bioquest Inc) dye dilution after serial stimulation with tumor cells. Briefly, CAR-T cells were incubated with CytoTell™ Red 650 dye at 37°C for 30 min, and the dye working solution was removed. Then, the CAR-T cells were co-cultured with tumor cells at a ratio of 1:1 or without tumor cells (no stimulation). After 3 days of co-culture, the proliferation of CAR-T cells was analyzed by flow cytometry, and the experiments were repeated for serial three rounds of antigen stimulation.

### IL-2-Dependent Proliferation Assay

We cultured 1 × 10^6^ RA^+^ CAR-T or RA^−^ CAR-T cells in the presence or absence of IL-2 (final concentration; 100 IU/ml) in 24-well cell culture plates. IL-2 was supplemented weekly, and the numbers of live cells were determined every 7 days.

### RNA-Sequencing and Bioinformatics Analysis

Total RNA was isolated from RA^+^ CAR-T cells and RA^−^ CAR-T cells after 14 days culture (these CAR-T cells were not sorted for CAR-positive population), with or without antigen stimulation, by co-culturing these cells with REH cells at an effector:target ratio of 1:1 for 3 days (RA^+^/Stimulation^+^, RA^−^/Stimulation^+^, RA^+^/Stimulation^−^, and RA^−^/Stimulation^−^, respectively), using RNeasy Mini Kit (Qiagen, Venlo, Netherlands). The concentration of total RNA was measured using NanoDrop 2000 (Thermo Fisher Scientific). Library preparation and high-throughput sequencing were performed using Eurofins Genomics (Ebersberg, Germany). In brief, mRNAs were enriched and their strand-specific library was prepared. Sequencing was performed using a NextSeq 500/550 system (Illumina, San Diego, CA, USA) and NextSeq 500/550 Mid Output Kit v2.5 150 cycles (Illumina). Adapter sequences and low-quality reads were removed using fastp version 0.21.0 (28). Filtered reads were aligned to the human reference genome (GRCh38.p13) using STAR version 2.7.6a (29). Counts per gene and transcripts per million were calculated using RSEM version 1.3.3 (30). Calculation of counts per million and differential expression analysis were performed using edgeR version 3.32.0 R package (31) and R v4.0.3 environment (https://www.R-project.org/). Pathway analysis was performed using R package for the Reactome Pathway Analysis (32). Differential gene expression profiles between RA^+^ and RA^−^ CAR were also analyzed and visualized using Morpheus (https://software.broadinstitute.org/morpheus) and specific gene signatures related to T cell activation, exhaustion, and differentiation (33).

### *In Vivo* Stress Test Using a Murine Systemic Tumor Model

Female 8-week-old NOD.Cg-*Prkdc^scid^Il2rg^tm1Wjl^*/SzJ (NSG) mice were purchased from Jackson Laboratory (Bar Harbor, ME, USA), and housed at the Kyoto Prefectural University of Medicine for more than a week before starting the experiment. Food and water were provided *ad libitum*. REH-FFLuc-GFP cells suspended in D-PBS were infused into mice *via* the tail vein. Six days later, RA^+^ CAR-T, RA^−^ CAR-T, or irrelevant CAR-T cells, which redirected the EPHB4 receptor (17) as a control, were infused *via* the tail vein, and tumor burdens were monitored using the IVIS Lumina Series III system (PerkinElmer, Inc.). The regions of interest on the displayed images were quantified in photons per second (ph/s) using Living Image v2 (PerkinElmer, Inc.) as described previously (17). Bone marrow (BM) cells were obtained by sequential BM aspiration from tibias at several time points. The BM cells were stained with a PE-conjugated anti-human CD3 antibody and APC-conjugated anti-human PD-1 antibody (BioLegend), and the long-term persistence of human T cells was evaluated by flow cytometry. The mice were euthanized at predefined endpoints, under conditions that met the euthanasia criteria given by the Center for Comparative Medicine at the Kyoto Prefectural University of Medicine.

### Statistics

Statistical comparisons between two groups were determined by two-tailed parametric or non-parametric (Mann–Whitney *U*-test) tests for unpaired data or by two-tailed paired Student’s *t*-test for matched samples. All data are presented as mean ± standard deviation. The log-rank test was used to compare survival curves obtained using the Kaplan–Meier method. A P-value of <0.05 was considered statistically significant. All the statistical analyses were performed using the GraphPad Prism 9 software.

## Results

### RA^+^ CAR-T Cells Exhibited Superior Transduction Efficiency and Expansion Capacity, Dominant CD8 Expression, Enriched Stem Cell Memory Fraction, and Lower Expression of Exhaustion-Related Markers Than RA^−^ CAR-T Cells

**Figure 1:**
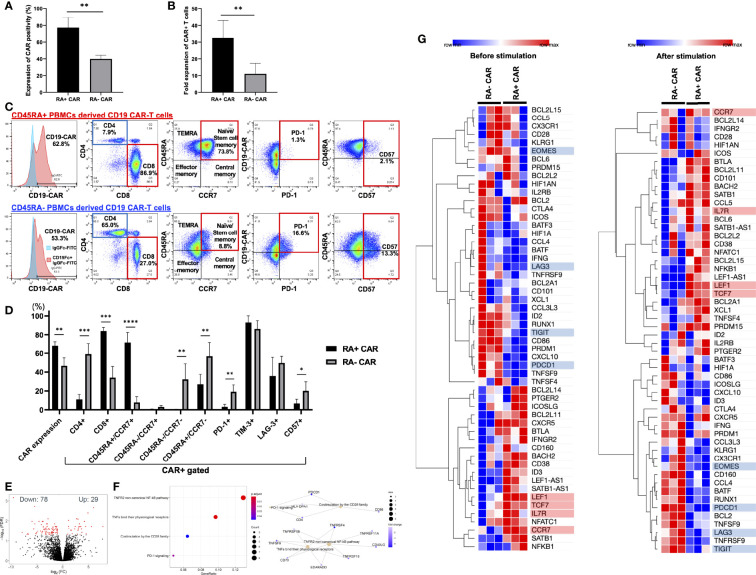
CD45RA^+^ chimeric antigen receptor (CAR)-T cells showed better transient gene transfer efficiency and expansion capacity, dominant CD8 expression, enriched stem cell memory fraction, and less expression of exhaustion-related markers than CD45RA^−^ CAR-T cells. **(A)** The transient expression of CAR transgene 24 h after gene transfer (n = 3, 3 donors). **(B)** Number of CAR positive T cells at day 14 (n = 5, 3 donors). **(C)** Representative expression and phenotypes of CD19 CAR-T, and expression of exhaustion markers on CAR-T cells assessed by flow cytometry. The gating control (left, blue) for CAR expression showed CAR-T cells only, stained by anti-human IgG Fc fragment specific antibody conjugated to FITC, the gating of CAR expression (left, red) showed CAR-T cells combined with the recombinant human CD19 Fc chimera protein and secondary stained by anti-human IgG Fc fragment specific antibody conjugated to FITC. **(D)** The phenotype and exhaustion marker of CD19 CAR-T cells are represented (n = 3–6, 3 donors). **(E)** A volcano plot showing genes with adjusted FDR <0.05 that are differentially expressed in CD45RA^+^ CAR-T compared with CD45RA^−^ CAR-T. **(F)** Reactome pathway analysis showed that several gene pathways were significantly downregulated in CD45RA^+^ CAR-T cells compared with CD45RA^−^ CAR-T. Both red and blue circles showed downregulation of gene pathways in CD45RA^+^ CAR-T, and their colors represented adjusted p-values. **(G)** Transcriptome profiling about expression of T-cell activation genes (highlighted by red underline) and exhaustion genes (highlighted by blue underline) in CD45RA^+^ CAR-T and CD45RA^−^ CAR-T, before antigen stimulation (left) and after antigen stimulation (right). Row min denotes lowest Z-score and row max denotes highest Z-score. All data are presented as means ± standard deviation. *P < 0.05, **P < 0.01, ***P < 0.001, ****P < 0.0001.

First, we isolated CD45RA^+^ or CD45RA^−^ subpopulations from whole PBMCs using human CD45RA-targeted magnetic separation. We observed two peaks of CD45RA high and negative populations in the lymphocyte fraction (**Supplementary Figure S2A**). By magnetic bead sorting, CD45RA^+^ and CD45RA^−^ PBMCs were efficiently separated with >98% of CD45RA positive cells in the CD45RA^+^ fraction and >93% of CD45RA negative cells in the CD45RA^−^ fraction (**Supplementary Figure S2A**). Both RA^+^ and RA^−^ PBMCs consisted of about 60% CD3-positive cells, and the rest of CD3-negative cells were positive for CD56 in RA^+^ PBMCs, while CD56, CD14 and CD15 were negative in RA^−^ PBMCs (**Supplementary Figure S2B**). Moreover, electroporation did not greatly influence the CD3 positivity of both RA^+^ and RA^−^ PBMCs (**Supplementary Figure S2C**). Indeed, RA^+^ and RA^−^ PBMCs both contained approximately 40% CD3-negative cells, which included NK cells and other myeloid cells, but this percentage of these cells decreased at 24 h after electroporation and more decreased after 14 days. Based on this observation, CD3-negative T cells were likely destroyed during electroporation since the optimized protocol for introducing the DNA plasmid required a relatively high voltage. We then evaluated the transient gene transfer efficiency of CD19 CAR transgene into CD45RA^+^ or CD45RA^−^ PBMCs 24 h after electroporation. When the CD19 CAR transgene plasmid was introduced into unstimulated, magnetically sorted CD45RA^+^ or CD45RA^−^ PBMCs by electroporation, CD45RA^+^ PBMC subpopulation exhibited higher transient gene transfer efficiency than CD45RA^−^ PBMC subpopulation 24 h after electroporation (**Figure 1A**). Furthermore, CD19 CAR-T cells derived from CD45RA^+^ PBMCs (RA^+^ CAR-T) exhibited higher expansion capacity 14 days after culture compared with CD19 CAR-T cells derived from CD45RA^−^ PBMCs (RA^−^ CAR-T) (**Figure 1B**). After 14 days of expansion, we determined the CAR positivity, phenotype, and exhaustion-related marker PD-1 expression on these CAR-T cells by flow cytometry. RA^+^ CAR-T cells consisted of about 95% CD3-positive cells and a small number of CD3-negative/CD56-positive NK cells, while RA^−^ CAR-T cells consisted of about 85% CD3-positive cells and about 13% CD3-negative/CD56-positive cells, suggesting that residual NK cells could affect the immune function of these populations (**Supplementary Figure S2D**). Compared with RA^−^ CAR-T, RA^+^ CAR-T cells exhibited higher CAR positivity, lower expression of exhaustion-related marker PD-1 and T-cell senescence marker CD57 (34, 35), and enriched naïve/stem cell memory fraction, which were associated with the longevity of CAR-T cells (**Figures 1C, D**). The copy number of the integrated CAR transgene was calculated by qPCR, and these CAR-T cells had ~20 copies of the CAR transgene (16.5 ± 1.1 in RA^+^ CAR-T cells, 5.9 ± 0.3 in RA^−^ CAR-T cells, and 20.2 ± 1.3 in CD19 CAR-T cells derived from whole PBMCs (**Supplementary Figure S3B**)), which was consistent with the previous report (11). Furthermore, RA^+^ CAR-T cells were markedly CD8-dominant, whereas the CD4/8 ratio of both RA^+^ and RA^−^ PBMCs, the starting material, was CD4-dominant. These results suggest that CD8-positive stem cell-like T cells are primarily amplified in RA^+^ CAR-T cells (**Supplementary Figure S1B**). By contrast, RA^−^ CAR-T cells are CD4-dominant in the final product, which may be detrimental to CAR-T cell function as RA^−^ CAR-T cells may harbor more regulatory CD4 CAR-T cells. Other activation/exhaustion-related markers such as TIM-3 and LAG-3 were highly expressed in both the types of CAR-T cells (**Figure 1D**), and this finding is consistent with that of previous studies on PB-CAR-T cells (14, 16–18, 36). As most PB-CAR-T cells were engineered by electroporation, we hypothesized that the high expression of TIM-3 and LAG-3 would be induced by the stimulation of electroporation. However, PBMCs 48 h after electroporation barely expressed PD-1, TIM-3, or LAG-3 on T cells (**Supplementary Figure S3**). Therefore, the expression of TIM-3 and LAG-3 but not PD-1 on CAR-T cells was induced during the incubation period and not by the stimulation of electroporation.

To further investigate the effect of RA^+^ CAR-T or RA^−^ CAR-T cells at the molecular level and to identify the pathways involved in the favorable phenotype of RA^+^ CAR-T cells, we performed genome-wide transcriptional profiling by focusing on immunogenic gene signatures. A total of 29 genes were identified with higher expression and 78 genes with lower expression in RA^+^ CAR-T cells compared with CD45RA^−^ CAR-T cells (**Figure 1E**). Reactome pathway analysis showed that the differential gene expression profiles observed in RA^+^ CAR-T cells were related to the non-canonical NF-κB pathway and co-stimulation by the CD28 family pathway; PD-1 signaling pathway was significantly downregulated in RA^+^ CAR-T cells (**Figure 1F**). Transcriptome profiling showed that RA^+^ CAR-T cells exhibited activated but less exhausted profiles characterized by the upregulation of T-cell activation-like markers including transcription factor 7 (TCF7), lymphoid enhancer-binding factor 1 (LEF1), CCR7, and IL7R and the downregulation of canonical exhaustion-related markers including PD-1, T-cell immunoreceptor with Ig and ITIM domains (TIGIT), and Eomesodermin (EOMES) in RA^+^ CAR-T cells (33) (**Figure 1G**, left), even after antigen stimulation (**Figure 1G**, right). LAG3 expression was also downregulated in RA^+^ CAR-T cells, but flow cytometry results did not show statistical differences between RA^+^ and RA^−^ cells (**Figure 1D**). These gene expression analysis data suggest that RA^+^ CAR-T cells have an abundant naïve/memory phenotype even when stimulated by antigen-positive tumor cells, corroborating the phenotype of RA^+^ CAR-T cells assessed by flow cytometry.

### Analysis of the Function of CAR-T Cells Using the *In Vitro* Serial Tumor Challenge Assay

**Figure 2:**
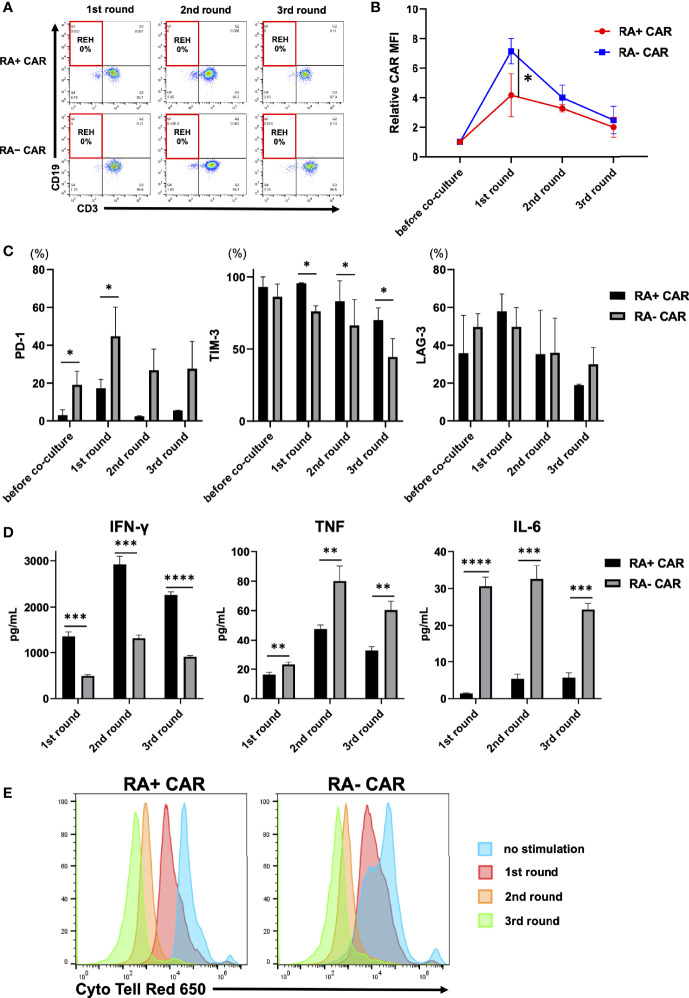
Analysis of chimeric antigen receptor (CAR) function by *in-vitro* serial tumor challenge assay. **(A)** The expression of CAR-T and REH cells during sequential co-culture. Representative dot plots are shown. **(B)** Relative CAR mean fluorescence intensity (MFI) (MFI = 1, before co-culture) of CAR-T cells during sequential co-culture (n = 3). CAR MFI of CAR-T cells were calculated after gating on the CAR positive population. **(C)** The expression of PD-1, TIM-3, and LAG-3 on CAR-T cells during sequential co-culture (n = 3). **(D)** The level of cytokines in the co-culture supernatant containing CAR-T cells with REH cells after sequential co-culture (n = 3). **(E)** Cell division of CAR-T cells upon repeated REH cells stimulations or no stimulation. All data are presented as means ± standard deviation. *P < 0.05, ***P < 0.01, ***P < 0.001, ****P < 0.0001. PD-1, programmed cell death protein-1; TIM-3, T cell immunoglobulin mucin-3.

To evaluate the antileukemic activity of RA^+^ CAR-T and RA^−^ CAR-T cells, we performed the tumor re-challenge assay in which fresh REH cells were added to CAR-T cells every three days. Both the types of CAR-T cells achieved complete killing of REH cells even after multiple rounds of tumor re-challenge (**Figure 2A**). Interestingly, the expression of CAR molecules on the cell surface after antigen stimulation in RA^+^ CAR-T cells was controlled at a relatively lower level than that in RA^−^ CAR-T cells (**Figure 2B**), which suggested less tonic CAR signaling and exhaustion of RA^+^ CAR-T cells compared with RA^−^ CAR-T cells (37, 38). PD-1 expression in RA^+^ CAR-T cells was lower than that in RA^−^ CAR-T cells during multiple rounds of antigen stimulation (**Figure 2C**). In contrast, the expression of LAG-3 was similar in RA^+^ CAR-T and RA^−^ CAR-T cells, whereas TIM-3 expression in RA^+^ CAR-T cells was higher than that in RA^−^ CAR-T cells; these expressions gradually decreased during multiple rounds of antigen stimulation in RA^+^ CAR-T and RA^−^ CAR-T cells, and relatively high expression of TIM-3 and LAG-3 did not impair the killing efficacy of CAR-T cells (**Figure 2C**). We also evaluated the production of inflammatory cytokines in the cell culture supernatant in response to serial co-culture with tumor cells. The secretion of IFN-γ was higher in RA^+^ CAR-T cells than in RA^−^ CAR-T cells, although transcriptome profiling showed that IFNG expression has been already downregulated in RA^+^ CAR-T cells on day 3 of co-culture with tumor cells (**Figure 1G**). Interestingly, the levels of TNF and IL-6 were significantly lower in RA^+^ CAR-T cells than in RA^−^ CAR-T cells (**Figure 2D**). Furthermore, the proliferation of RA^+^ CAR-T and RA^−^ CAR-T cells during serial stimulation was evaluated by CytoTell™ dye dilution with tumor cells. Although RA^−^ CAR-T cells proliferated faster than RA^+^ CAR-T cells without stimulation, there was no significant difference between the proliferation of RA^+^ CAR-T cells and that of RA^−^ CAR-T cells after serial tumor stimulation (**Figure 2E**). Notably, the proliferation of both RA^+^ and RA^−^ CAR-T cells did not occur spontaneously, but was dependent on antigen stimulation or cytokine supplementation, as confirmed by an IL-2-dependent proliferation assay (**Supplementary Figure S4**), indicating no disordered proliferative potential in these cells.

### RA^+^ CAR-T Cells Showed Better Tumor Control With Long-Term Expansion of CAR-T Cells Than RA^−^ CAR-T Cells or Unsorted CAR-T Cells in *In Vivo* Stress Test

**Figure 3:**
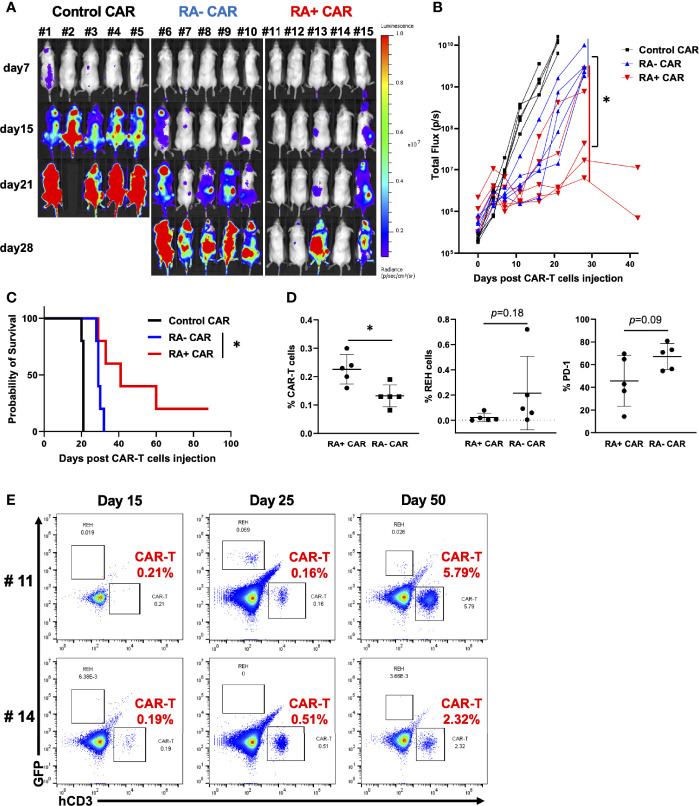
CD45RA^+^ chimeric antigen receptor (CAR)-T cells achieved prolonged tumor control with long-term expansion of CAR-T cells *in vivo.* We infused 5 × 10^5^ REH-FFLuc cells into NSG mice *via* the tail vein. Six days later, 1 × 10^5^ RA^+^ CAR-T, RA^−^ CAR-T, or control (EPHB4) CAR-T positive cells were infused into the tail vein of each mouse. **(A)** Bioluminescence images of groups of five NSG mice after intravenous CAR-T cell infusion. **(B)** The tumor volumes of each mouse measured as total flux (p/s) are shown. The CD45RA^+^ CAR-T group showed a statistically significant tumor reduction, measured as the mean total flux at day 28, compared with the CD45RA^−^ CAR-T group. **(C)** The Kaplan–Meier plot of overall survival (each group, n = 5). The CD45RA^+^ CAR-T group achieved prolonged tumor control compared with the CD45RA^−^ CAR-T group. Log-rank test: *P < 0.05. **(D)** On day 15 after CAR-T cell injection, bone marrow analysis showed CAR-T cells (left), REH cells (middle), and PD-1 expression on CAR-T cells (right) by flow cytometry. **(E)** In most long-lived mice infused with RA^+^ CAR-T cells, CAR-T cells, and REH cells in the bone marrow of mice on days 15, 25, and 50. Representative dot plot data are shown. All data are presented as mean ± standard deviation. *P < 0.05.

To evaluate the *in vivo* antitumor efficacy of RA^+^ CAR-T and RA^−^ CAR-T cells, we performed the *in vivo* stress test in which CAR-T cell dosage was lowered to the functional limits, so that these CAR-T cells were maintained and expanded *in vivo* to achieve antitumor efficacy. Because CAR expression in RA^+^ CAR-T and RA^−^ CAR-T cells was not exactly the same, we injected 1 × 10^5^ CAR-positive T cells in each group. RA^+^ CAR-T cells induced greater tumor reduction and prolonged median survival than those of RA^−^ CAR-T cells (**Figures 3A–C**). On day15, bone marrow in the RA^+^ CAR group exhibited abundant human CD3 positive T cells with lower expression of PD-1 and a relatively smaller number of REH cells than that in the RA^-^ CAR group (**Figure 3D**). Furthermore, in two of the long-lived mice in the RA^+^ CAR group, human CD3^+^ T cells were expanded even after 50 days of treatment as confirmed by sequential bone marrow studies (**Figure 3E**), which indicated antigen-induced proliferation and long-term functionality of RA^+^ CAR-T cells *in vivo*. The gating strategy of the bone marrow study was shown in **Supplementary Figure S5**.

**Figure 4:**
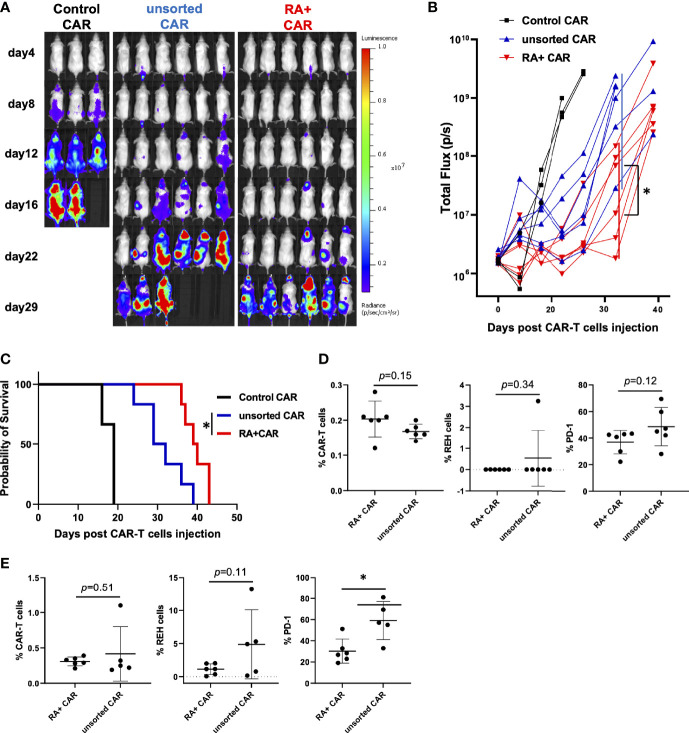
CD45RA^+^ chimeric antigen receptor (CAR)-T cells achieved prolonged tumor control than unsorted CAR-T cells *in vivo* stress test. We infused 1 × 10^6^ REH-FFLuc cells into NSG mice *via* the tail vein. Six days later, 1 × 10^5^ RA^+^ CAR-T (n = 6), unsorted CAR-T (n = 6), or control (EPHB4) CAR-T (n = 3) positive cells were infused into the tail vein of each mouse. **(A)** Bioluminescence images of groups of NSG mice after intravenous CAR-T cell infusion. **(B)** The tumor volumes of each mouse measured as total flux (p/s) are shown. The CD45RA^+^ CAR-T group showed a statistically significant tumor reduction, measured as the mean total flux at day 22, compared with the unsorted CAR-T group. **(C)** The Kaplan–Meier plot of overall survival. The CD45RA^+^ CAR-T group achieved prolonged tumor control compared with the unsorted CAR-T group. Log-rank test: *P < 0.05. **(D)** On day 15 after CAR-T cell injection, bone marrow analysis showed CAR-T cells (left), REH cells (middle), and PD-1 expression on CAR-T cells (right) by flow cytometry. **(E)** On day 25 after CAR-T cell injection, bone marrow analysis showed CAR-T cells (left), REH cells (middle), and PD-1 expression on CAR-T cells (right) by flow cytometry. All data are presented as mean ± standard deviation. *P < 0.05.

To evaluate whether the selection of CD45RA^+^ PBMCs as a starting material would facilitate highly effective PB-CAR-T cell manufacturing, we performed the *in vivo* stress test in RA^+^ CAR-T and PB-CD19 CAR-T from unsorted (both CD45RA^+^ and CD45RA^−^) PBMCs (unsorted CAR-T). We infused 1 × 10^6^ REH-FFLuc cells into NSG mice *via* the tail vein, and six days later, 1 × 10^5^ CAR-positive T cells were infused into each group. RA^+^ CAR-T cells achieved greater tumor reduction and prolonged median survival than unsorted CAR-T cells (**Figures 4A–C**). Sequential bone marrow studies in the RA^+^ CAR group showed abundant human CD3 positive T cells with lower expression of PD-1 on day 15 (**Figure 4D**), lower expression of PD-1 in human CD3 positive T cells, and a relatively smaller number of REH cells than the unsorted CAR group (**Figure 4E**).

## Discussion

In this study, we generated PB-CD19 CAR-T cells using magnetically-isolated CD45RA^+^ and CD45RA^−^ PBMCs as the starting materials. We found that CD45RA^+^ PBMCs were susceptible to the introduction of CAR transgene by electroporation, and RA^+^ CAR-T cells exhibited superior expansion capacity than RA^−^ CAR-T Cells. RA^+^ CAR-T cells were less vulnerable to T-cell exhaustion-related markers by multiple antigen stimulation, as evidenced by genome-wide transcriptome profiling, and RA^+^ CAR-T cells demonstrated prolonged tumor control than RA^−^ CAR-T cells or even bulk CAR-T cells from unsorted PBMC in the *in-vivo* stress test. Therefore, CD45RA-positive PBMC selection from PBMCs starting from PB-CAR-T cell manufacturing would be important to improve the efficacy of the therapy.

Tonic-antigen stimulated or antigen-independent early T-cell exhaustion greatly impairs the function of CAR-T cells. Previous studies have shown that retrovirally-engineered CAR-T cells exhibit strong *in vitro* killing activity; however, the cells exhaust soon and fail to control tumor growth (38–40). In the clinical setting, differentiation and exhaustion profiles of T cells in the final products are associated with their clinical response and antitumor efficacy (8, 9). Various modifications reported in CAR-T engineering to prevent early T-cell exhaustion are as follows: use of endogenic promoters for stable expression of the CAR transgene (37), use of a combination of internal ribosome entry site constructs into the CAR transgene to reduce CAR-transgene expression (40), or modification of immunoreceptor tyrosine-based activation motifs in the CD3ζ chain (41). The PB-transposon system is an effective and adaptable tool for transgene delivery as an alternative for viral vectors, not only because of its cost-effectiveness and simple T-cell engineering process (42) but also the preferred T-cell phenotype and less exhausted profiles (16–18). Indeed, the present study and other studies have reported that PB-CAR-T cells exhibit a memory-rich CAR-T cell phenotype regardless of the target antigen or the manufacturing procedure; these CAR-T cells long-lived when infused in the tumor-bearing murine model and exhibited prolonged antitumor potency (17, 18). In the present study, PB-transgenes were preferentially introduced into CD45RA^+^ PBMCs, and the memory-rich CD19 CAR-T cells were preferably enriched by PB-based manufacturing, which would be associated with the long-term functionality of PB-CAR-T cells. Indeed, IFN-γ in RA^+^ CAR-T cells was greatly increased by antigen stimulation, and thus IFN-γ reached a high level 3 days after stimulation (**Figure 2D**). However, the activation of RA^+^ CAR-T cells was transient and soon normalized, as evidenced by the downregulation of the IFN gene in RA^+^ cells (**Figure 1G**). These results suggest that RA^+^ CAR-T cells are not over-activated by antigen stimulation which would be related to the lower expression of immune exhaustion-related markers and long-term functionality.

PD-1 is an important exhaustion-related marker expressed on CAR-T cells that limits their function (43); CAR-T cell therapy combined with PD-1 blockade can be a potential strategy in cancer treatment (44–47). In this study, RA^+^ CAR-T cells scarcely expressed PD-1, even after multiple rounds of antigen stimulation. In contrast, other canonical exhaustion-related markers such as TIM-3 and LAG-3 were highly expressed in both RA^+^ and RA^−^ CAR-T cells, although their expression gradually decreased during antigen stimulation. Genome-wide transcriptome profiling showed that RA^+^ CAR-T cells, despite of highly expressed TIM-3 and LAG-3, exhibited enriched expression of memory-T cell-like genes and less exhausted profiles. TIM-3 was initially identified as a molecule expressed by dysregulated, chronically-activated T cells and is generally considered to be a T-cell inhibitory protein (48, 49). However, the recent studies have indicated that TIM-3 exerts paradoxical costimulatory effect on T cells, including enhancement of the phosphorylation of ribosomal S6 protein, which is present downstream of T-cell receptor signaling (50, 51). We did not investigate the co-stimulatory function of TIM-3 on PB-CAR-T cells; however, based on the *in vitro* and *in vivo* potency of TIM-3 positive RA^+^ CAR-T cells, the high expression of TIM-3 and LAG-3 on PB-CAR-T cell surface might not induce exhaustion which could impair the function of PB-CAR-T cells.

The expression profiles of several cell surface molecules are associated with the memory phenotype. We selected CD45RA as the marker for starting material selection, because clinical-grade CD45RA (or CD45RO) selection has been established and already used in the clinical setting (52). Therefore, the isolation of CD45RA^+^ PBMC from the leukapheresis product could be easily translated into the clinical setting for PB-CAR-T cell manufacturing. Nevertheless, other memory-related T-cell surface molecules-positive PBMCs, such as CCR7 or CD62L-positive PBMCs, may also be potential starting materials for PB-CAR-T cell manufacturing. Moreover, previous studies have reported that a specific formulation of post-manufactured CAR-T cell products would enhance antitumor efficacy (53); however, the cellular preparation of starting materials or post-manufacturing products has not been optimized yet. Nevertheless, CD45RA-positive selection of starting PBMCs would be beneficial in reducing the risk of manufacturing failure, which sometimes occurs in the clinical setting.

In a recent clinical trial conducted in Australia, two patients developed PB-mediated CAR-T cell-derived lymphoma (54). Although the researchers did not find transgene incorporation into known oncogenes and could not identify a unifying pathogenic mechanism, they determined a relatively high CAR transgene copy number in the malignancies and insertion of the CAR transgene into the BACH2 locus in both malignancies. The PB transposase they used was called “hyperactive PB”, which was engineered to have a higher incorporation capacity and therefore would be related to the insertional mutagenesis. In the present study, we used an originally-developed PB transposase (24), and the copy number of the CAR transgene was relatively low compared to data from patients who developed lymphoma in a previous clinical trial (54). Nevertheless, there have been a number of clinical trials of CAR-T cells using hyperactive PB systems, but no malignant transformation has been reported except in a recent trial (54). It is also possible that the incorporation profile of CAR transgene may affect the malignant transformation. We did not evaluate the integration profile of CAR-T cells on a genome-wide basis, which is a limitation of our study. Similar to genetic manipulation by retroviruses, PB-mediated genetic modification is executed non-randomly in terms of integration sites, such as by favoring integration near the transcription start site (24). In fact, a previous report demonstrated that the transgene profile of PB-mediated CAR-T cells generated by the exactly the same PB system that we used is similar to that of clinically accepted retroviral CAR-T cells (55). These suggested that not only the PB system but also the entire manufacturing processes, including plasmid and manufacturing reagents, or the patients background, might have contributed to malignant transformation in the previous clinical trial (54). Therefore, the safety of genetically modified T cells, including the long-term toxicity, should be thoroughly evaluated before clinical application.

In conclusion, PB-mediated RA^+^ CAR-T cells exhibited a memory-rich phenotype and superior antitumor function *in vivo*, thereby indicated that the selection of CD45RA^+^ PBMCs as a starting material would be useful for efficient PB-CAR-T cell manufacturing. The development of clinical grade and automatic cell isolation technologies may further facilitate genetically modified T-cell engineering with greater functionality and simplicity.

## Data Availability Statement

The datasets presented in this study can be found in online repositories. The names of the repository/repositories and accession number(s) can be found in the article/**Supplementary Material**.

## Ethics Statement

The studies involving human participants were reviewed and approved by the Kyoto Prefectural University of Medicine Institutional Review Board. The patients/participants provided their written informed consent to participate in this study. The animal study was reviewed and approved by the Kyoto Prefectural University of Medicine Institutional Review Board.

## Author Contributions

Conceptualization, SY. Methodology, MS, SY, NN, SK, YS, and MT. Investigation, MS, SY, NN, SK, and YS. Writing, Original Draft, MS and SY. Writing, Review & Editing, MS, SY, and TI. Funding Acquisition, SY. Resources, SY and TI. Supervision, SY, YN, and TI. All authors contributed to the article and approved the submitted version.

## Funding

This work was supported by the Japan Agency for Medical Research and Development (AMED) (18ck0106413h0001) and the JSPS KAKENHI (17K07224).

## Conflict of Interest

Authors NN, SK, YS were employed by the company AGC Inc.

The remaining authors declare that the research was conducted in the absence of any commercial or financial relationships that could be construed as a potential conflict of interest.

## Publisher’s Note

All claims expressed in this article are solely those of the authors and do not necessarily represent those of their affiliated organizations, or those of the publisher, the editors and the reviewers. Any product that may be evaluated in this article, or claim that may be made by its manufacturer, is not guaranteed or endorsed by the publisher.
