# Supplementary figures and images for: PiggyBac Transposon-Mediated CD19 Chimeric Antigen Receptor-T Cells Derived From CD45RA-Positive Peripheral Blood Mononuclear Cells Possess Potent and Sustained Antileukemic Function
Source: Front Immunol. 2022 Jan 27;13:770132. doi: 10.3389/fimmu.2022.770132 (PMC8829551; doi:10.3389/fimmu.2022.770132)

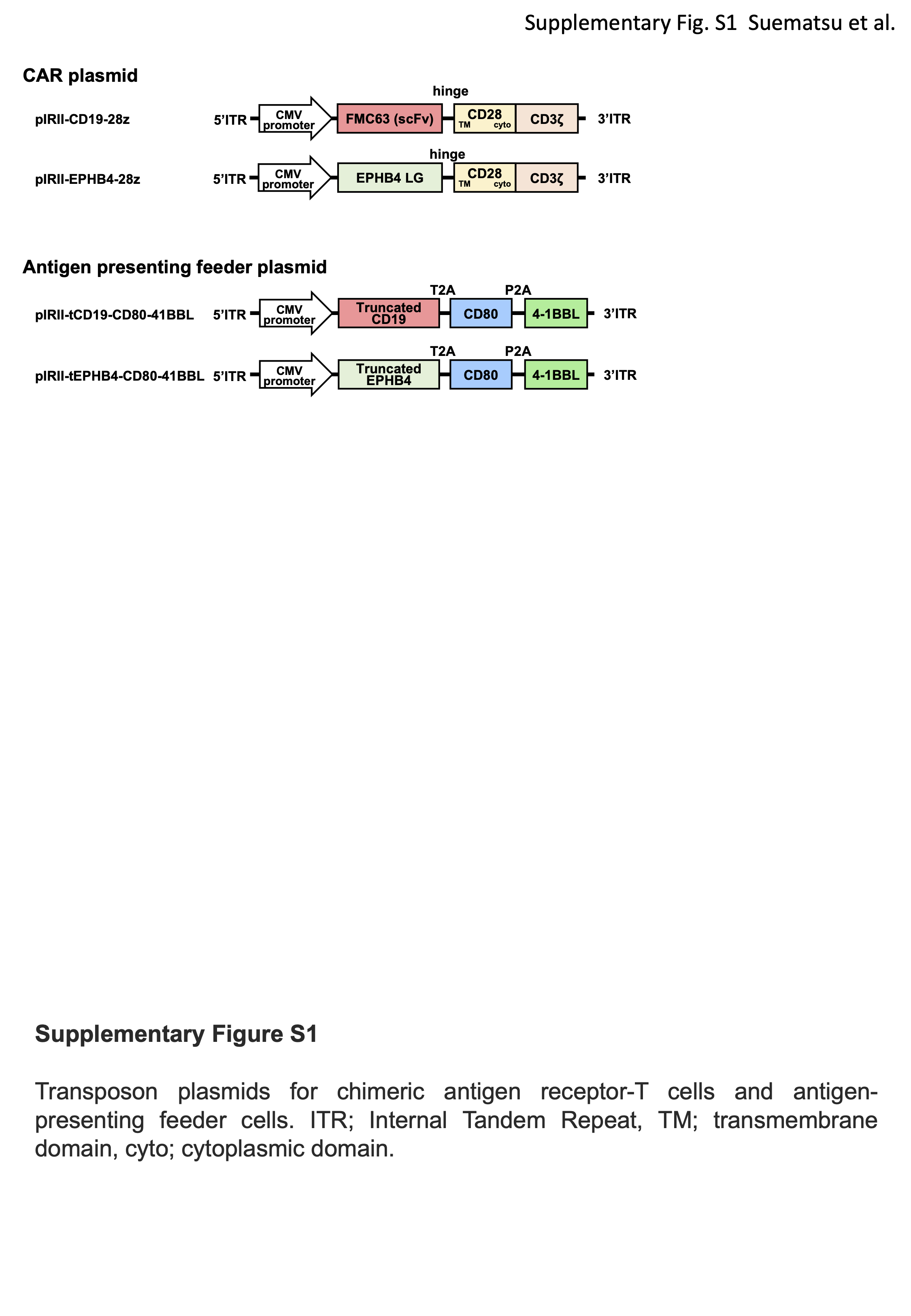

Supplement: Supplementary file 1 [file Image_1.tiff]

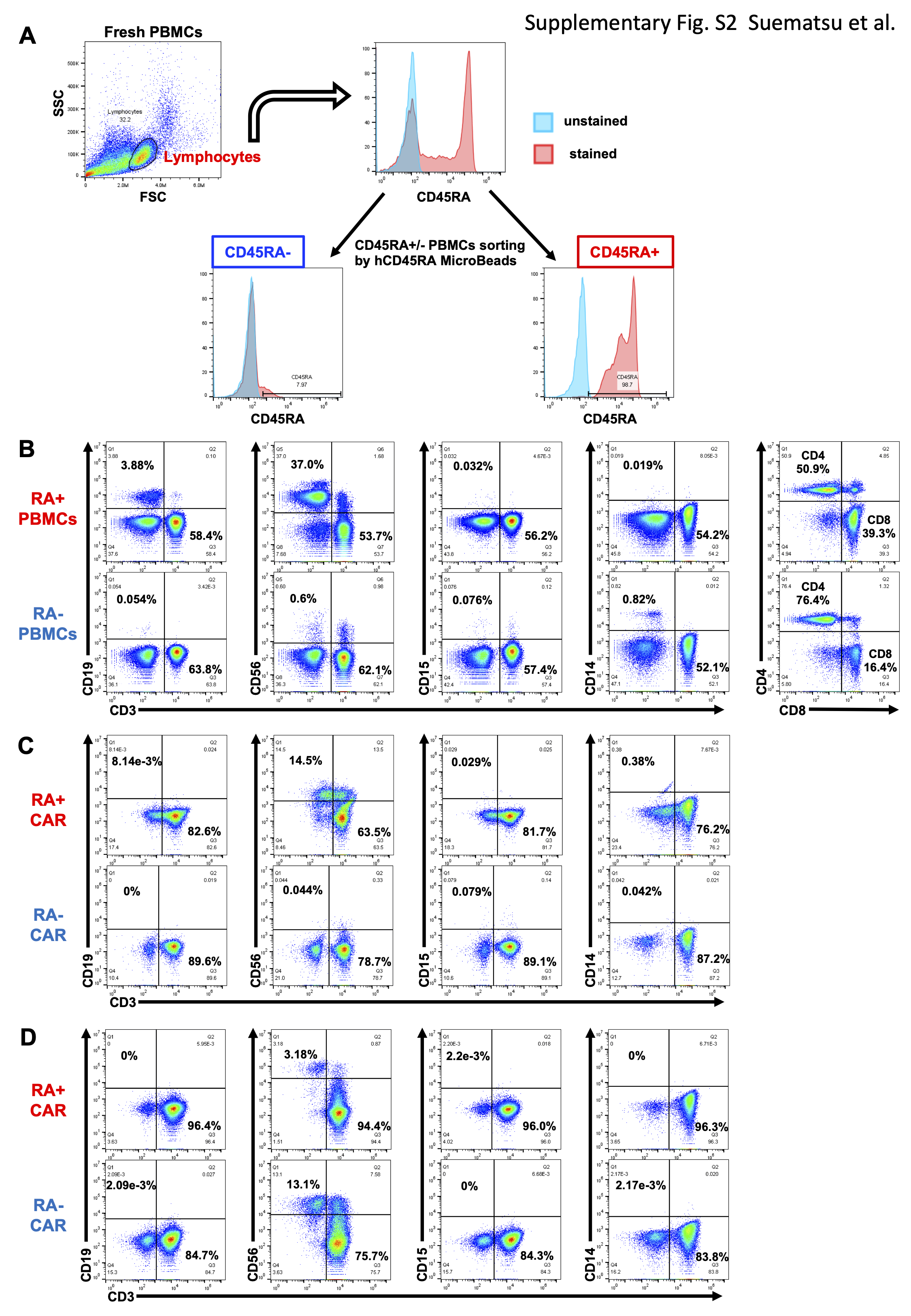

Supplement: Supplementary file 2 [file Image_2.tiff]

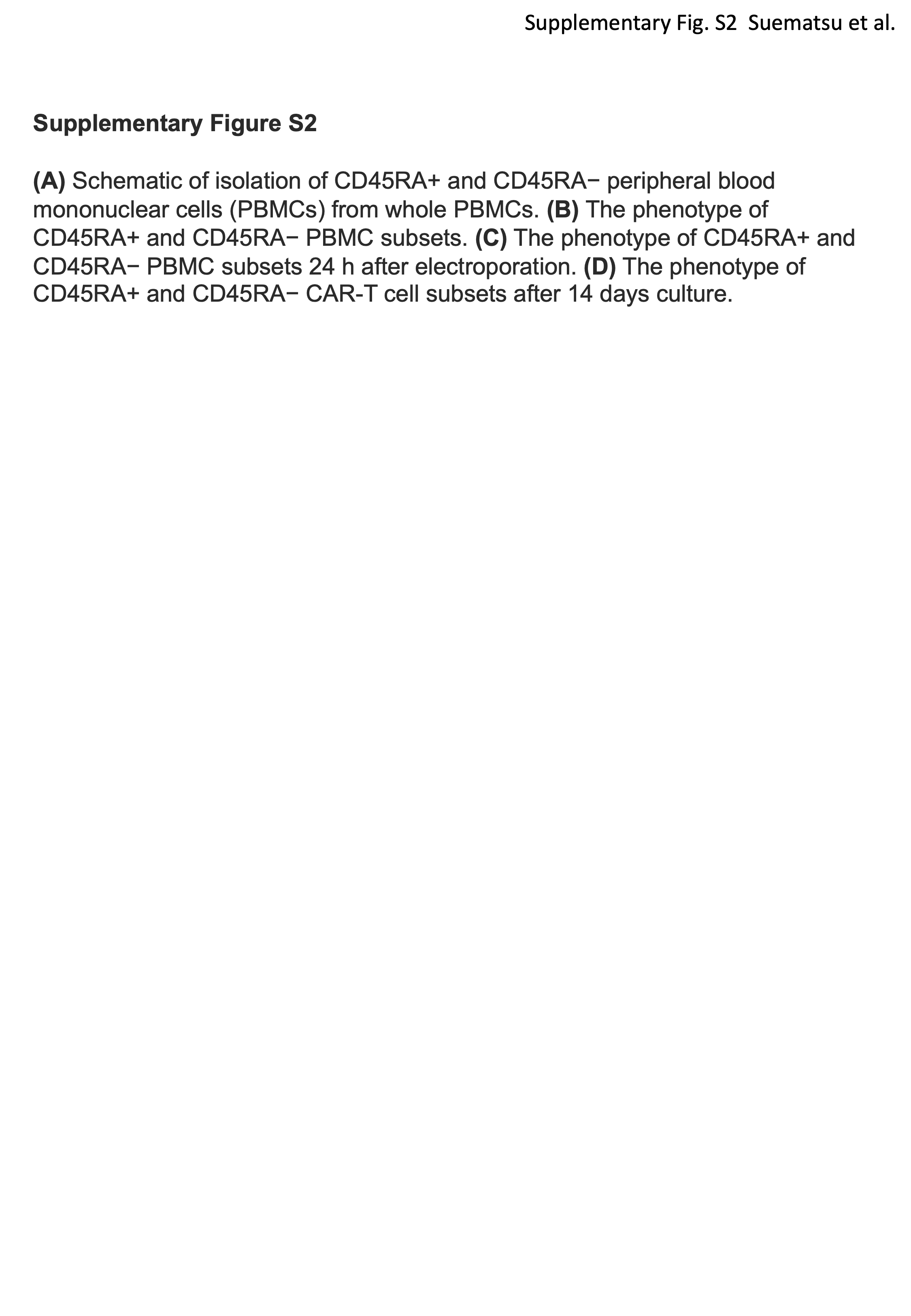

Supplement: Supplementary file 3 [file Image_3.tiff]

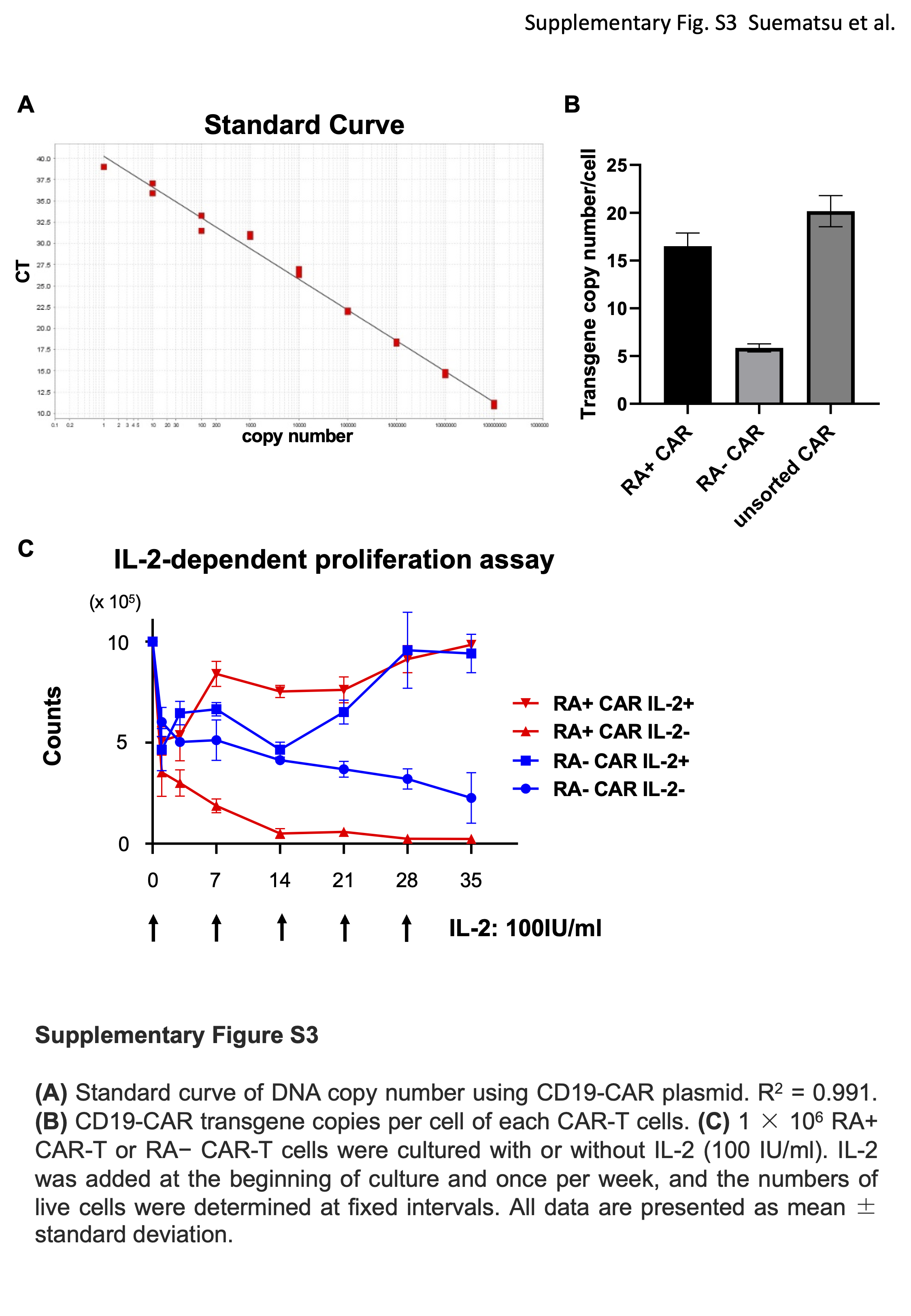

Supplement: Supplementary file 4 [file Image_4.tiff]

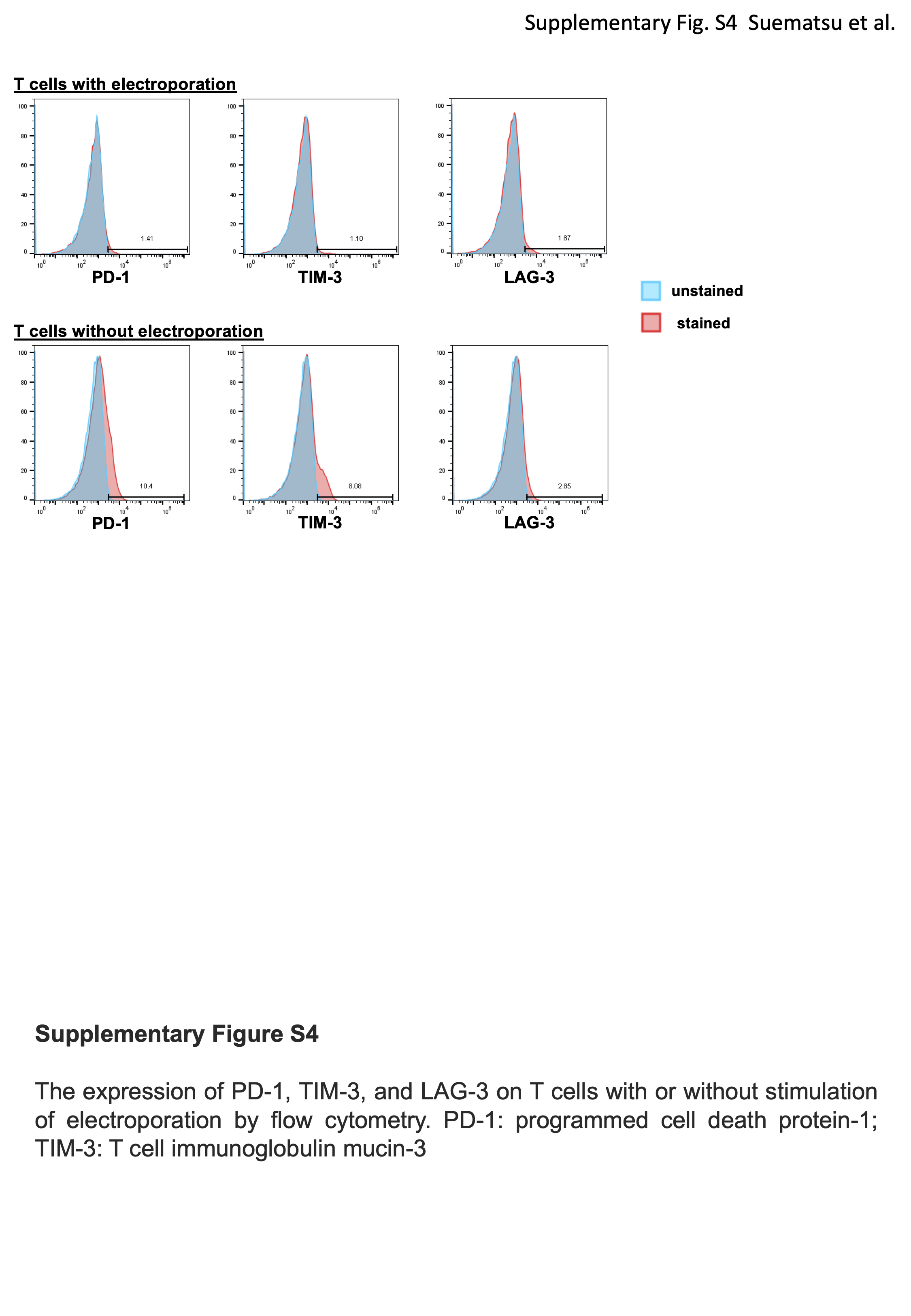

Supplement: Supplementary file 5 [file Image_5.tiff]

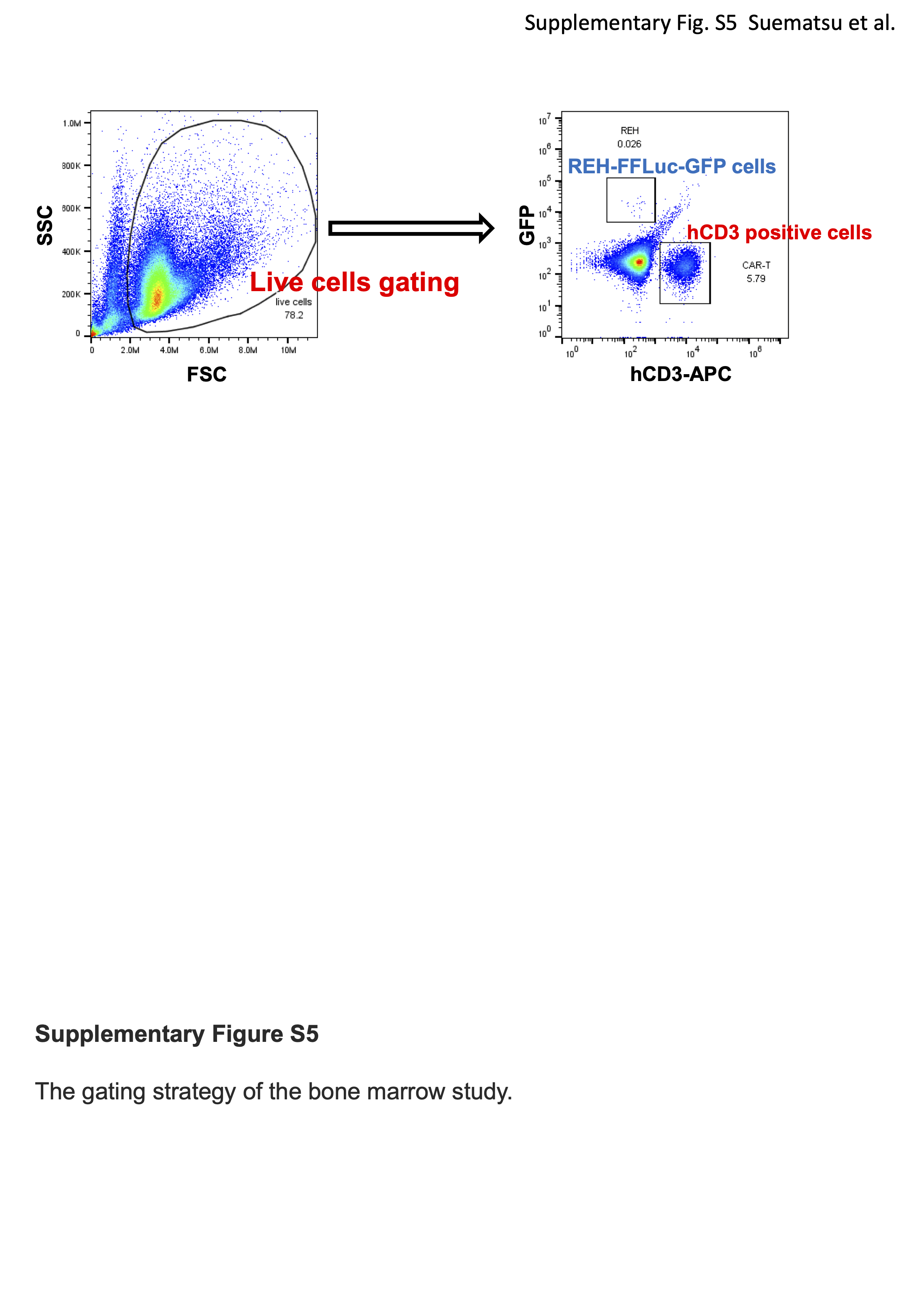

Supplement: Supplementary file 6 [file Image_6.tiff]

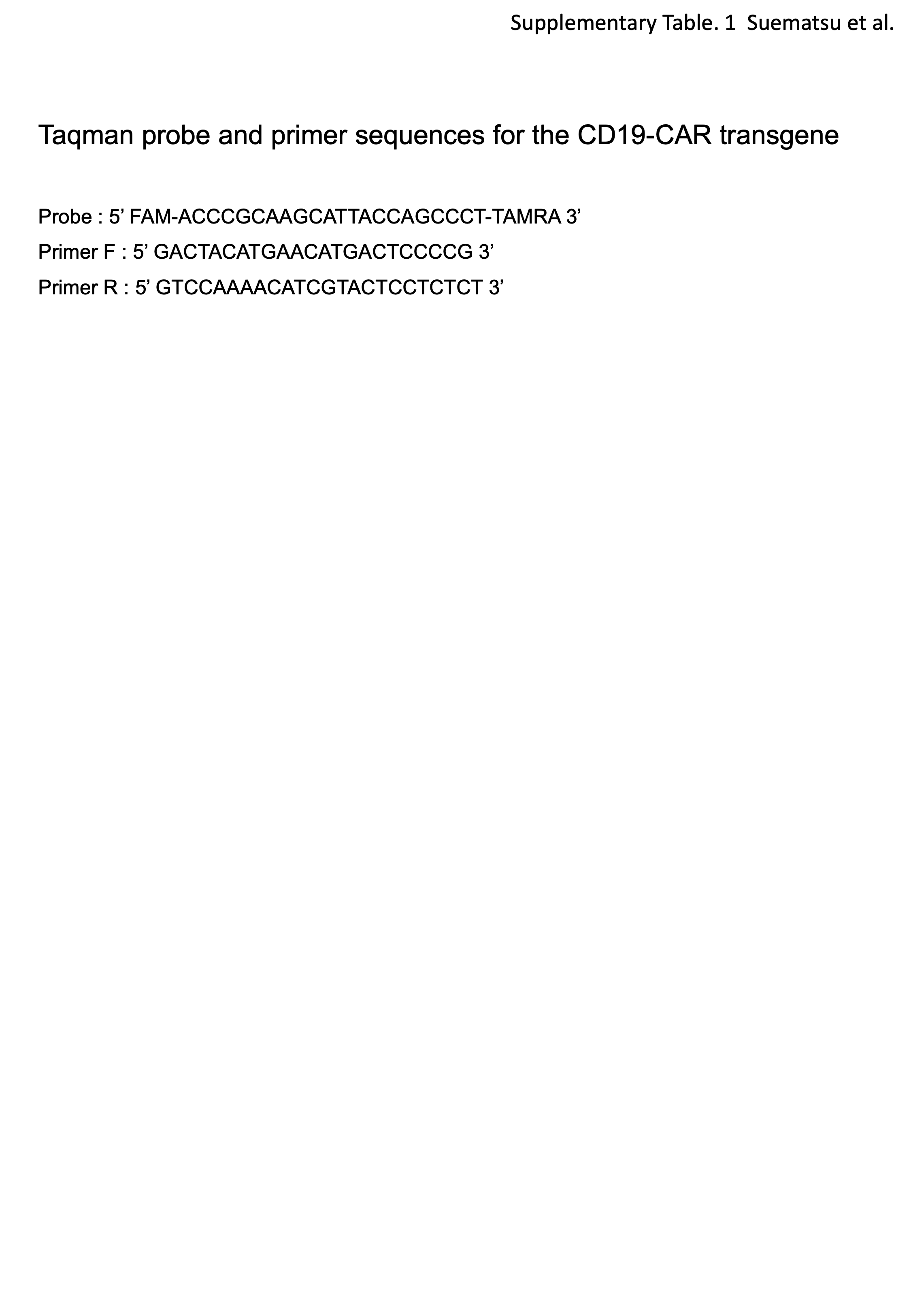

Supplement: Supplementary file 7 [file Image_7.tiff]
